# Role of bone morphogenetic protein-7 in renal fibrosis

**DOI:** 10.3389/fphys.2015.00114

**Published:** 2015-04-23

**Authors:** Rui Xi Li, Wai Han Yiu, Sydney C. W. Tang

**Affiliations:** Division of Nephrology, Department of Medicine, The University of Hong Kong, Queen Mary Hospital, Hong KongChina

**Keywords:** BMP-7, chronic kidney disease, renal fibrosis, cytokines, inflammation

## Abstract

Renal fibrosis is final common pathway of end stage renal disease. Irrespective of the primary cause, renal fibrogenesis is a dynamic process which involves a large network of cellular and molecular interaction, including pro-inflammatory cell infiltration and activation, matrix-producing cell accumulation and activation, and secretion of profibrogenic factors that modulate extracellular matrix (ECM) formation and cell-cell interaction. Bone morphogenetic protein-7 is a protein of the TGF-β super family and increasingly regarded as a counteracting molecule against TGF-β. A large variety of evidence shows an anti-fibrotic role of BMP-7 in chronic kidney disease, and this effect is largely mediated via counterbalancing the profibrotic effect of TGF-β. Besides, BMP-7 reduced ECM formation by inactivating matrix-producing cells and promoting mesenchymal-to-epithelial transition (MET). BMP-7 also increased ECM degradation. Despite these observations, the anti-fibrotic effect of BMP-7 is still controversial such that fine regulation of BMP-7 expression *in vivo* might be a great challenge for its ultimate clinical application.

## Introduction

Renal fibrosis, which characterized as glomerulosclerosis and tubulointerstitial fibrosis, is considered the hallmark of progressive renal injury and the final common pathway of multiple chronic renal diseases. Regardless of the primary causes, appearance of most “end stage kidney” manifested as extensive scar formation (fibrotic tubules and sclerotic glomeruli), thickened arteries, and infiltrated chronic inflammatory cells. Process of renal fibrosis involved activation of intrinsic kidney cells, infiltrated cells, and led to excessive accumulation and deposition of extracellular matrix and finally the loss of kidney function. TGF-β has been known as the key modulator of kidney (and that of other organs) fibrosis, and the role of TGF-β in renal fibrosis has been extensively studied. In recent years, another protein, BMP-7, that belongs to the TGF-β superfamily, has drawn great attention for its function in counteracting pro-fibrotic effects of TGF-β. BMP-7 is a homodimeric protein that with cysteine-knot. It plays an crucial role in renal development and only selectively expressed in several adult organs including the kidney. It is a natural negative regulator of nephrotic TGF-β/Smad signaling pathway. The fact that renal BMP-7 disappears during renal fibrogenesis (Wang et al., 2001; Morrissey et al., 2002; Yang et al., 2007) and, supply of BMP-7 either exogenously or endogenously results in prevention or even reversal of functional and structural changes of various nephropathies in animal models (Vukicevic et al., 1998; Hruska et al., 2000; Wang et al., 2003, 2006; Zeisberg et al., 2003a,b; Chan et al., 2008), strongly promises a renal protective function of BMP-7 in human kidney disorders. And its anti-fibrotic role in kidney disease has been extensively studied. A number of review articles have summarized the possible mechanisms and potential therapeutic targets of renal fibrosis (Liu, 2006; Boor et al., 2010; Eddy, 2014; Kawakami et al., 2014; Lee et al., 2014; Munoz-Felix et al., 2014). This review focuses on role of BMP-7 in fibrotic kidney disease, and the possible mechanisms involved.

## Individual sections

### Mechanisms of renal fibrosis

Fibrosis can be considered as ineffective wound healing process, in which excessive progression of scarring rather than resolution occurs. Fibroblasts play a pivotal role in the process, which is modulated by multiple cell types and molecules. In this respect, the cellular and molecular response of the damaged kidney attempted to prevent renal damage and preserve renal function, and as a matter of fact, almost all cell types of the kidney and a series of infiltrating cells are involved in the development of renal fibrosis, indicating the complexity of this process.

In early phase of renal injury, damage cells release cytokines and chemokines, which attract inflammatory cells infiltrated into injured sites (You and Kruse, 2002). Although inflammatory response is an important portion of the body defense mechanism, the non-resolving inflammation always become major driving force of fibrogenic process (Nathan and Ding, 2010; Schroder and Tschopp, 2010). The process of renal fibrosis is almost always accompanied with infiltration of inflammatory cells including macrophages, mast cells, lymphocytes, neutrophil as well as dendritic cells. After injury, the infiltrated inflammatory cells activated and release more chemotactic cytokines, vasoactive factors and increase production of profibrotic factors (Ferenbach et al., 2007; Ricardo et al., 2008; Vernon et al., 2010; Chung and Lan, 2011), thus sustained a profibrotic microenvironment in injured sites, and interacted with kidney intrinsic cells, which finally caused phenotypic and functional changes of kidney cells, increase the number of myofibroblast and ECM formation (Duffield, 2014; Mack and Yanagita, 2015). Therefore, persistent inflammation which was caused by injury trigger the onset of profibrotic stage and serve to sustain the profibrotic pressure in the tissue (Meng et al., 2014).

The sustained increase of profibrotic cytokines in microenvironment after renal injury inevitably leads to activation of matrix-producing cells, which plays a central role in renal fibrosis. Fibroblasts are considered as the major source of ECM in the kindney, besides, other cell types in renal tubulointerstitium such as tubular epithelial cells, vascular smooth muscle cells and macrophages are also capable of producing ECM. Myofibroblasts are terminally differentiated cells that rarely found in normal renal tissue. It functions for interstitial ECM synthesis and accumulation including collagen I, collagen III, fibronectin during wound healing or scar forming at injured sites. There are at least five source of myofibroblasts have been proposed in experimental kidney models or disease kidneys, including activation of endogenous fibroblasts (which was regarded as classical source of myofibroblasts), differentiation of pericytes, tubular epithelial to mesenchymal transition, bone marrow fibroblast infiltration or endothelial phenotypic transition (Duffield, 2014). Regardless of their origin, the presence of myofibroblasts are predictor of fibrotic progression, once detectable, they are prognostic indicators of fibrotic expansion. Intervening the activity of myofibroblasts and preventing their accumulation in the kidney has become a therapeutic target of antifibrotic treatment.

Activated fibroblasts from all sources produce large amounts of ECM components, leading to excessive accumulation and deposition of interstitial matrix, which is predominantly composed of collagen I, III, and fibronectin. In the physiological condition, the production and degradation of ECM is intensely regulated under a dynamic balance. Under the profibrotic state, this delicate balance is interrupted. Activation of profibrotic factors and inactivation of antifibrotic factors tilt the balance, increasing ECM formation while reducing their degradation, finally leading to excessive accumulation of ECM. Key fibrogenic factors include TGF-β1, connective tissue growth factor (CTGF), angiotensin II, platelet-derived growth factor (PDGF) and fibroblast growth factor (FGF)2, among which, TGF-β1 has been regarded as key modulator of renal fibrosis (Bottinger, 2007), and the role of TGF-β in fibrosis has been extensively studied and well summarized (Branton and Kopp, 1999; Bottinger, 2007; Lan, 2011).

Transforming growth factor-β (TGF-β) is a multifunctional cytokine, functioned through its cognate receptors (type II and I) to activate a variety of canonical and non-canonical signaling pathways with large amount of profibrotic target genes (Bottinger, 2007). Although most intrinsic kidney cells can secret and respond to TGF-β, fibroblasts and myofibroblasts are particularly responsive to TGF-β stimulation. TGF-β1 mediates renal fibrogenesis by increasing ECM production and reducing degradation, besides, TGF-β1 is strong stimulant to induce phenotypic transformation of epithelial cells into mesenchymal cells (EMT) (Lan, 2003; Bi et al., 2012; Moustakas and Heldin, 2012). Blockade of TGF-β1 by neutralizing antibodies or gene silencing, decorin have demonstrated to reduce ECM production and ameliorate renal fibrosis in both *in vitro* and *in vivo* studies (Border and Noble, 1998), whereas overexpression of TGF-β1 in mouse liver develop progressive liver and renal fibrosis (Sanderson et al., 1995; Kopp et al., 1996). Studies targeted on TGF-β signaling have been done by many research groups, mainly focusing on reducing TGF-β production or blockade of TGF-β signal transduction. Clinical trials have also been done to evaluate efficacy of anti-TGF-β antibodies in fibrotic diseases (Mead et al., 2003; Denton et al., 2007), while results were less promising as anticipated. Anti-TGF-β treatment with pirfenidone, an orally active small molecule that inhibit TGF-β through reducing promoter activity and protein secretion, showed beneficial effect in diabetic mouse model (RamachandraRao et al., 2009) and in human focal segmental glomerulosclerosis (FSGS) (Cho et al., 2007), a recently completed placebo-controlled randomized clinical trial also demonstrated the efficacy of pirfenidone on improving GFR in overt DN (Sharma et al., 2011). However, due to the multiple pathophysiological functions of TGF-β, systemic administration of anti- TGF-β antibodies may have significant side effect given that mice knockout of TGF-β1 developed chronic inflammation in almost all organs (Boivin et al., 1995), and knockout of TGF-β2 die soon after their birth (Sanford et al., 1997). Therefore, strategies also target on TGF-β signaling regulation including TGF-β receptor blockade, receptor Smad regulation or other downstream protein control.

CTGF is a cytokine with a molecular weight around 36–38 kD, it is thought to be an prominent profibrotic downstream modulator of TGF-β (Leask and Abraham, 2006). Under pathological circumstances like fibrotic diseases or skin scarring, overexpression of CTGF was observed (Leask and Abraham, 2004). Elevated of constitutive CTGF is a hallmark of tissue firbosis, it acts as co-factor of ECM, growthfactor and cytokines which created an permissive enviroment for other stimuli to induce profibrotic response (Leask and Abraham, 2006). Plasma level of CTGF was independent predictor of ESRD extend and the overall mortality (Nguyen et al., 2008b). Blockade of CTGF by using antisense oligonucleotides or specific down-regulation with interfering RNA (siRNA) ameliorates renal tubulointerstitial fibrosis and the progression of nephropathy (Yokoi et al., 2004; Guha et al., 2007). Phase I clinical trial of anti-CTGF monoclonal antibody FG-3019 in type 1 and 2 diabetic patients was well tolerated and associated with a decrease in albuminuria (Adler et al., 2010).

### Efficacy of BMP-7 as a therapeutic drug in renal fibrosis

BMP-7, formerly called osteogenic protein-1/OP-1, is a member of the BMP-subfamily within the transforming growth factor β (TGF-β) superfamily. BMP-7 expression in normal kidney has the highest expression level in adult organs (Dudley et al., 1995; Luo et al., 1995). Under several disease states like ischemia-reperfusion injury (Simon et al., 1999), diabetic nephropathy (Wang et al., 2001), and hypertensive nephrosclerosis (Bramlage et al., 2010), BMP-7 has been reported to be down-regulated in the kidney, and increased again during the regenerative phase (Vukicevic et al., 1998; Simon et al., 1999). Hypothesis that BMP-7 possesses anti-fibrotic activity was first verified in a rat model of unilateral ureteral obstruction (UUO) (Hruska et al., 2000), based on previous observations that BMP-7 was a renal morphogen (Dudley et al., 1995), such that its loss and re-expression pattern mirrors preservation of renal function in acute renal injury (Vukicevic et al., 1998). The simplified model of renal interstitial fibrosis was generated by UUO, in which renal injury was mediated partly through activation of the angiotensin II -TGF-β cascade (El-Dahr et al., 1993; Diamond et al., 1994; Yoo et al., 1997; Fern et al., 1999). Exogenous administration of recombinant human BMP-7 blocked tubular epithelial cell apoptosis, reduced tubular cells dropped out and the accumulation of ECM. This observation suggested a convincing anti-fibrotic effect of BMP-7 in renal interstitial fibrosis.

Shortly after this observation, researchers sought to explore the role of BMP-7 in a rat model of STZ-induced diabetes, which resembles human type 1 diabetes. BMP-7 was given exogenously through i.v. injection. In this model, it was found that BMP-7 administration delayed the onset of diabetic nephropathy and prevented glomerulosclerosis; and even partially reversed diabetic kidney hypertrophy and restored GFR in the progression stage of DN (Wang et al., 2003). Subsequent studies also demonstrated a protective role of BMP-7 in STZ-induced diabetic kidney injury, manifesting as reduced urinary protein excretion, preserved podocyte nephrin expression (Xiao et al., 2009). In another STZ-induced diabetic model, the CD1 mouse, which tends to develop more severe and accelerated diabetic kidney injury, BMP-7 inhibited glomerular lesion and tubulointerstitial fibrosis (Sugimoto et al., 2007). Besides exogenously supplemented BMP-7, transgenic overexpression of BMP-7 in FVB/N mice induced with STZ led to reduced podocyte dropout, glomerulosclerosis and interstitial collagen accumulation (Wang et al., 2006).

The beneficial effects of BMP-7 are not just restricted to DN. In MRL/MpJ background and autoimmune disease-prone MRL/MpJ*^lpr/lpr^* mice, which developed chronic renal injury resembling lupus nephritis, BMP-7 dose dependently reduced interstitial ECM protein accumulation, tubular atrophy, serum creatinine and improved prognosis (Zeisberg et al., 2003a). In addition, BMP-7 improved kidney morphology and renal function in Col4A3 knockout mice that recapitulate Alport Syndrome, a genetic kidney disease. CD1 mice treated with nephrotoxic serum to induce acute glomerulonephritis had decreased ECM secretion and even prompted mesenchymal to epithelial transition when BMP-7 was administered (Sugimoto et al., 2007).

Among these studies, a prominent anti-fibrotic effect of BMP-7 in both glomerulus and tubulointerstitium was observed. Nevertheless, considering that BMP-7 was first discovered as growth factor that facilitated bone formation, concerns about ectopic bone formation and vascular calcification of using BMP-7 systemically seem reasonable. To date, no reports suggested these potential complications in their rodent models. Furthermore, there was evidence that BMP-7 even prevented vascular calcification (Davies et al., 2005; Hruska et al., 2005). Transgenic mice that over-expressed BMP-7 for 1 year had no observable ectopic bone formation either (Wang et al., 2006).

However, BMP-7 was not always effective and promising. For example, BMP-7 conferred no benefits in a rat protein-overload model (Ikeda et al., 2004). In this study, BMP-7 showed no effect in reducing ECM accumulation and proteinuria; on the contrary, BMP-7 tended to increase ECM gene expression in the kidney. Another study investigated on LDLR-deficient mice with kidney partial ablation also showed no effect of BMP-7 in preserving renal function, though BMP-7 corrected hyperphosphatemia, osteodystrophy and prevented vascular calcification (Davies et al., 2005). Most published studies of BMP-7 as a therapeutic intervention in animal models of kidney disease were listed in Table 1. Furthermore, clinical trials of BMP-7 analog THR-V-123 has been launched by Thrasos to study its efficacy on diabetic nephropathy, which results worth anticipated.

**Table 1:** **Published studies of BMP-7 as a therapeutic tool in animal models of kidney disease**.

**Animal model**	**Species**	**Disease model**	**Effect of BMP-7**	**References**
IRI	Rat	Acute tubular necrosis	Preserved kidney function, increase survival rate	Vukicevic et al., 1998
UUO	Rat	Obstructive nephropathy	Preserved renal blood flow, prevent tubular atrophy, reduced tubulointerstitial and fibrosis	Hruska et al., 2000
MRL/MpJ*^lpr/lpr^*	Mouse	Lupus nephritis	Inhibited tubular atrophy and interstitial fibrosis	Zeisberg et al., 2003a
COL4A3−/−	Mouse	Alport syndrome	Inhibited tubular atrophy and interstitial fibrosis	Zeisberg et al., 2003a
Nephrotoxic serum nephritis	CD1 Mouse	Acute glomerulonephritis	Induced MET, decreased ECM secretion	Zeisberg et al., 2003b, 2005
STZ induced diabetes	Rat	Type 1 diabetic nephropathy	Reversed kidney hypertrophy, restored GFR, reduced albumin excretion	Wang et al., 2003
Kidney Cx or 5/6 Nx (RRKM)	SD rat	CKD	Increased tubular regeneration in early stage of repair process	Dube et al., 2004
STZ induced diabetes	CD1 Mouse	Type 1 diabetic nephropathy	Inhibit glomerular lesion, and tubulointerstitial fibrosis	Sugimoto et al., 2007
STZ induced diabetes	FVB/N Mouse	Type 1 diabetic nephropathy	Reduced podocytes dropout, glomerular fibrosis and interstitial collagen accumulation	Wang et al., 2006
LDLR−/−	Mouse	CKD/VC/Atherosclerosis/	Down regulated osteocalcin expression	Davies et al., 2003
Unx + kidney partial ablation LDLR−/−	Mouse	Metabolic syndrome/Insulin resistance	Corrected hyperphosphatemia, osteodystrophy, and prevent VC, but no improvement of renal function	Davies et al., 2005
STZ induced diabetes	Rat	Type 1 diabetic nephropathy	Reduced urine protein excretion, preserved podocyte number and nephrin expression	Xiao et al., 2009
Protein overload	Rat	Kidney disease	Failed to reduced proteinuria and even showed increase of ECM gene expression	Ikeda et al., 2004

*IRI, Ischemia-reperfusion injury; UUO, unilateral ureteral obstruction; STZ, streptozotocin; Unx, uninephrectomy; CKD, chronic kidney disease; VC, vascular calcification; Kidney Cx, decapsulation; 5/6 Nx, 5/6 nephrectomy; RRKM, rat remnant kidney model*.

### Mechanism of anti-fibrotic effect of BMP-7

#### BMP-7 and TGF-β

TGF-β has been known as the key modulator of kidney (and that of other organs) fibrosis. TGF-β acts on multiple cell types in the kidney, including podocytes, mesangial cells, renal proximal tubular epithelial cells and interstitial fibroblasts. TGF-β induces pro-fibrotic gene transcription, and finally leads to the overexpression of extracellular matrix proteins, reduction of cell proliferation and differentiation, and elevation of resident epithelial cell apoptosis. Moreover, although evidence of epithelial-to-mesenchymal transition (EMT) in human kidney remains insufficient, TGF-β have been shown to super-induce fibrotic progression in dozens of *in vitro* studies of tubular epithelial cells, by causing epithelial phenotypic lost and gain of fibroblast phenotypes. As a member of the TGF-β superfamily, BMP-7 was first speculated to promote fibrosis progression in the kidney, but interestingly, an abundance of researches concluded an opposite view.

Besides the anti-fibrotic effect of BMP-7 in all the animal models mentioned above, a variety of *in vitro* studies have suggested the anti-fibrotic effect of BMP-7 to be heavily (although may not exclusively) TGF-β-dependent. This raised the hypothesis that the anti-fibrotic effect of BMP-7 was mediated via counterbalancing the TGF-β signaling pathways. A mass of *in vitro* findings showed evidence to support this notion, and the interaction between BMP-7 and TGF-β in the kidney has also been discussed previously (Meng et al., 2013).

TGF-β induced extracellular matrix and other pro-fibrotic gene overexpression in mesangial cells. Although mesangial cells did not express BMP-7, BMP receptors were detectable on mesangial cells with active signals. Co-incubating murine mesangial cells with TGF-β and BMP-7 reduced TGF-β-stimulated collagen IV, fibronectin and CTGF overexpression. It is worth mentioning that BMP-7 reduces ECM accumulation without concomitant changes of their mRNA levels, suggesting that BMP-7 might act through affecting ECM protein degradation. Besides, TGF-β stimulation decreased matrix metalloprotease (MMP)-2 expression and this action could be abolished by BMP-7, possibly via reducing the activation of the plasminogen activator inhibitor (PAI-1) (Wang and Hirschberg, 2003). Furthermore, BMP-7 reduced nuclear accumulation of Smad3 and blocked the transcriptional up-regulation of CAGA-lux. Knock-down of Smad5 impaired the ability of BMP-7 to interfere with CAGA-lux activation (Wang and Hirschberg, 2004).

The effects of BMP-7 on tubular epithelial cells have also been extensively studied. The emerging importance of tubular epithelial cells (the most abundant cell type in the kidney) in kidney diseases cannot be overemphasized. Most studies in the animal models stated above mainly focused on tubulointerstitial damage, fueling attention of how BMP-7 functions on TECs.

Like mesangial cells, proximal tubular epithelial cells showed no expression of BMP-7, but constitutively express BMP receptors as described in the previously section. On the other hand, distal tubular cells express both BMP-7 and BMP receptors.

In a setting that study of BMP-7 on NP-1 cell (mouse distal tubule cell line) which stimulated with TGF-β (Zeisberg et al., 2003b), BMP-7 preserved epithelial cell phenotype and helped re-express E-cadherin and ZO-1 in NP-1 cells. They constructed a ligand-independent system with construct expressing ALK5 and a plasmid expressing Smad3 to mimic TGF-β intracellular signal, and a plasmid expressing Smad5 to mimic BMP-7. Mimicry of BMP-7 increased activity of E-cadherin promotor activity, while mimicry of TGF-β showed the opposite, suggesting Smad-dependent counteractions of TGF-β and BMP-7.

Researches have also been done in human proximal tubular epithelial cell line (HK2). BMP-7 dose dependently reduced TGF-β-induced overexpression of a-SMA, fibronectin, collagen I and CTGF in HK2 cells (Xu et al., 2009). Similar effects reproduced elsewhere by other group (Luo et al., 2010) with further research. BMP-7 was found to reduce Smad3 DNA binding to a consensus Smad binding element probe without alteration of Smad3 phosphorylation or degradation. Co-incubation with TGF-β revealed that BMP-7 reduced Smad3 binding to the PAI-1 promotor in HK2 cells. Moreover, BMP-7 prevented TGF-β-induced SnoN lost. siRNA interference suggested that the effect of BMP-7 on Smad3 was SnoN-dependent.

Cyclosporine A (CsA) has been reported to induce EMT in HK2 cells with involvement of TGF-β and CTGF (McMorrow et al., 2005). BMP-7 inhibited CsA-induced TGF-β and CTGF overexpression in a dose dependent manner (Xu et al., 2010). BMP-7 also reduced aristolochic acid (AA)-induced TGF-β and collagen 3 secretion, preserved cell phenotype and cell viability in HK-2 cells (Wang et al., 2010). These findings suggested a strong TGF-β-dependent anti-fibrotic role of BMP-7.

However, not all the studies with BMP-7 in TGF-β-induced fibrosis showed a positive result. Dudas et al., used TGF-β as a stimulant to study the role of BMP-7 in both human and murine tubular epithelial cells. Contrary to the other results listed above, they failed to demonstrate an anti-fibrotic role of BMP-7 in human tubular cells (both primary cell and immortalized cell line). BMP-7 *per se* decreased E-cadherin expression (Veerasamy et al., 2009) and increased vimentin, CTGF and TGF-β1 expression. However, in TCMK-1 cell (mouse renal tubular epithelial cell), BMP-7 preserved E-cadherin expression in TCMK-1 cells (Dudas et al., 2009), suggesting different modulatory role of BMP-7 in different species.

#### BMP-7 and CTGF

CTGF is considered as inhibitor of BMP-7 signaling. In diabetic CTGF+/+ mice, phosphorylation of smad1/5, expression of BMP-7 target gene Id1 and MMP activity was significantly lower than in CTGF+/− mice (Nguyen et al., 2008a). The secreted CTGF can directly binds to BMP and TGF-β through its CR domain, which prevent BMP binds to its receptors and contrarily enhance TGF-β-receptor binding (Abreu et al., 2002), by which, CTGF act as fibrogenic switch that shifting balance from anti-fibrosis to fibrosis (Gressner and Gressner, 2008). However, there are also evidences suggested BMP-7 alone increase gene expression of CTGF in tubular cells (Dudas et al., 2009; Li et al., 2015), which make interactions between BMP-7 and CTGF more complicated and pending for further investigation.

#### BMP-7 and ECM

Another possible hypothesis is that BMP-7 exerts anti-fibrotic effect by affecting ECM formation and degradation. BMP-7 reduced TGF-β induced overexpression of ECM proteins in mesangial cells without affecting their gene levels, besides, BMP-7 preserves MMP2 activity and blocks PAI-1 promoter activation in TGF-β-stimulated mesangial cells (Wang and Hirschberg, 2003), however, there were also studies suggested that MMP2 and -9 may play minor roles in ECM degradation (Holmbeck et al., 1999; Visse and Nagase, 2003; Lee et al., 2006). Study of BMP-7 in protein-overloaded rats showed a slight decrease of FN and collagen protein levels (not reaching statistical significance) but an increase of TGF-β gene expression, while in this study, endogenous BMP-7 expression was increased (Ikeda et al., 2004). Studies done by our group also observed increase of FN and collagen gene expression in PTECs that incubated with BMP-7. On the other hand, applied BMP-7 to human renal adult fibroblasts cell line reduced collagen I expression and promote cell mesenchymal-to-epithelial phenotypic transition (Zeisberg et al., 2005). Considering BMP-7 as a morphogen in bone formation, and the promotion of ECM composition was also reported in other study (Tacke et al., 2007) that increase of BMP-7 concentration was correlated with liver fibrosis. Thus, whether BMP-7 reduce or increase ECM remains controversial, there might be fine regulation of BMP-7 level *in vivo*, which may affected its role shifting between anti-fibrotic or pro-fibrotic.

#### BMP-7 and inflammation

Inflammatory response can be considered as the onset of wound healing, while non-resolution inflammation is a relentless driver of fibrogenesis that forms a vicious cycle to finally lead to irreversible fibrosis. Anti-inflammatory treatment on the onset and progression stage of renal fibrosis also attracted highly attention. Current studies of BMP-7 in the kidney mainly focused on its anti-fibrotic role, though many studies have suggested the possible anti-inflammatory effect of BMP-7. In the IRI model, BMP-7 reduced ICAM-1 expression and accumulation and activity of neutrophils (Vukicevic et al., 1998). Latter study with UUO model suggested BMP-7 reduced monocyte/macrophage infiltration in the tubulointerstitium (Hruska et al., 2000). Study that done by Stephen E Gould et al showed that co-incubated of TNF-α-stimulated human tubular cells with BMP-7 reduced overexpression of pro-inflammatory cytokines, including IL-6, IL-8, IL-1β, and MCP-1 (Gould et al., 2002). *In vitro* study with human mesangial cells suggested BMP-7 inhibited IL-1β-induced overexpression of MCP-1 expression in mesangial cells, which might exert by suppressing the JNK signaling pathways (Lee et al., 2003). BMP-7 also reduced overexpression of TNF-α, IL-6 that induced by polymeric IgA in mesangial cells (Chan et al., 2008). Moreover, there was study of BMP-7 on monocyte polarization, which found BMP-7 enhanced THP-1 polarized to M2 macrophage, and the increase of anti-inflammatory cytokines IL-10 and IL-1ra (Rocher et al., 2012). Our data also demonstrated BMP-7 reduced overexpression of proinflammatory cytokines in advanced glycation end products (AGEs)-stimulated PTECs (Li et al., 2015) and IgA-induced mesangial cells (Chan et al., 2008). This anti-inflammatory effect of BMP-7 might exert through suppressing activation of multiple signaling pathways including p38 and p44/42 MAPK, as well as reduction of ROS formation. Table 2 listed most published studies of BMP-7 as a therapeutic intervention in different cell types of the kidney.

**Table 2:** **Published studies of BMP-7 as a therapeutic tool in different cell types of kidney disease**.

**Cell type**	**Stimulants**	**Effect of BMP-7**	**References**
Murine podocyte	HG 25 mM TGF-β 100 pM	Reduced capase-3 activity, reduced apoptosis	Mitu et al., 2007
Murine podocyte	HG	Restored synaptopodin and podocin	De Petris et al., 2007
Rat mesangial cell	HG 30 nM	Decreased ROS and TGF-β	Yeh et al., 2009
Mesangial cell	polymeric IgA	Reduced expression of TNF-α, IL-6, TGF-β, fibronectin	Chan et al., 2008
Mesangial cell	TGF-β 50–200 pM	Reduced ECM accumulation, maintained activity of MMP2	Wang and Hirschberg, 2003
Mesangial cell	TGF-β 12.5–200 pM	BMP-7-induced opposition to TGF-β required Smad5	Wang and Hirschberg, 2004
Human PTECs	HSA 5 mg/ml	Repressed NIK dependent chemokine synthesis	Lim et al., 2014
HK2 cell	AA 30, 60, 120 umol/L	Preserved cell phenotype and cell viability, reduced TGF-β and collagen 3 secretion	Wang et al., 2010
HK2 cell	TGF-β1 ng/ml	Suppressed PAI-1, CTGF and TGF-β expression, preserved SnoN expression	Luo et al., 2010
HK2 cell	CsA 4.2 μM	Preserved epithelial cell phenotype, restored E-cadherin and decreased ECM secretion	Xu et al., 2010
HK2 cell	TGF-β3 ng/ml	Reversed EMT decreased ECM expression	Xu et al., 2009
HK2 cell and RPTEC and TCMK-1 cells	TGF-β 10 ng/ml (RPTEC); 3 ng/ml (HK2)	Failed to attenuate EMT in RPTEC or HK-2 cells, BMP-7 alone decreased E-cadherin expression and increased vimentin, CTGF and TGF-β1 expression; Preserved E-cadherin in TCMK-1 cells	Dudas et al., 2009
HK2 cell	MCP-1 0.1, 1, 10, 50 ng/ml	Inhibited MCP-1 induced EMT through TGF-β-Smad 3 pathways	Tan et al., 2005
TK173	N/A	Increased fibroblast E-cadherin, Pax2, Wnt4 expression and E-cadherin promoter activity (MET)	Zeisberg et al., 2005
HK2 cell	U937 cell condition media TNF-a 10 ng/ml	Reduced U937-dependent TGF-β promoter activity and TGF-β synthesis through interaction with cell surface hyaluronan;	Zhang et al., 2005
NP-1 cell	TGF-β 3 ng/ml	Preserved E-cadherin and ZO-1 expression	Zeisberg et al., 2003b

*TK173, human renal fibroblast cell line; NP-1, mouse distal tubular epithelial cells; HK2, human tubular epithelial cells; RPTEC, human primary tubular epithelial cells; TCMK-1, mouse renal tubular epithelial cells; HAS, human serum albumin; HG, high glucose; AA, Aristolochic*.

#### BMP-7 and oxidative stress

Role of oxidative stress in tissue fibrogenesis has been discussed extensively (Poli, 2000; Aragno et al., 2008; Zhao et al., 2008b; Nie and Hou, 2012). In the kidney, oxidative stress contributed to the onset and progression of renal fibrogenesis in different disease models (Zhang et al., 2004; Zhao et al., 2008a; Kim et al., 2009; Singh et al., 2011). On the other hand, previous studies have demonstrated that BMP-7 protected cultured neurons from oxidative stress, and BMP-7 reduced H_2_O_2_ toxicity to the neuron and lipopolysaccharide (LPS) stimulation (Tsai et al., 2007; Sun et al., 2011), which indicated an anti-oxidative role of BMP-7. Subsequently, Yeh et al. (2009) showed that BMP-7 decreased HG-induced ROS in mesangial cells, and that neutralizing BMP-7 with anti-BMP-7 or knockdown BMP-7 expression would increase superoxide generation in the cells. This antioxidative activity of BMP-7 might be exerted through suppressing JNK and c-jun phosphorylation and the reduction of PKCζ.

## Summary and perspectives

Increasing evidence from different independent experiments has approved the anti-fibrotic effect of BMP-7 in renal fibrotic disease regardless of its primary causes. BMP-7 exerts anti-inflammatory, anti-fibrotic and anti-oxidative responses caused by various stimuli, reduces ECM production by suppressing matrix-producing cell activity and inducing MET, enhances ECM degradation by increasing the activity of MMP and reducing activation of PAI-1. Clinical trials have also been launched to investigate the efficacy of BMP-7 (analog) in both acute and chronic kidney disease. However, despite the increasing understanding of BMP-7 signaling transduction and regulation, there are also controversial results in its efficacy. For instance, there was no effect of BMP-7 in skin, lung or renal fibrosis, and some studies even showed that BMP-7 is a promoter of fibrosis. Based on the overall results from all *in vitro* and *in vivo* studies to date, the anti-fibrotic effect of BMP-7 seems promising, whereas the diverse regulatory effect of BMP-7 in ECM protein and gene expression, and its interaction with profibrotic mediators like CTGF indicates that the precise role of BMP-7 could shift between anti-fibrosis or pro-fibrosis and the fine regulation of its level *in vivo* might be a major challenge in the systemic use of BMP-7 in clinical application.

## Author contributions

Rui Xi Li, Wai Han Yiu and Sydney C. W. Tang designed the study. Rui Xi Li and Wai Han Yiu carried out the experiments. Rui Xi Li analyzed the data and wrote the manuscript. Wai Han Yiu provided technical support. Sydney C. W. Tang edited the manuscript.

### Conflict of interest statement

The authors declare that the research was conducted in the absence of any commercial or financial relationships that could be construed as a potential conflict of interest.
